# Women Up, Men Down: The Clinical Impact of Replacing the Framingham Risk Score with the Reynolds Risk Score in the United States Population

**DOI:** 10.1371/journal.pone.0044347

**Published:** 2012-09-12

**Authors:** Matthew C. Tattersall, Ronald E. Gangnon, Kunal N. Karmali, Jon G. Keevil

**Affiliations:** 1 Division of Cardiovascular Medicine, Department of Medicine, University of Wisconsin School of Medicine and Public Health, Madison, Wisconsin, United States of America; 2 Department of Population Health Sciences, University of Wisconsin School of Medicine and Public Health, Madison, Wisconsin, United States of America; The University of Hong Kong, Hong Kong

## Abstract

**Background:**

The Reynolds Risk Score (RRS) is one alternative to the Framingham Risk Score (FRS) for cardiovascular risk assessment. The Adult Treatment Panel III (ATP III) integrated the FRS a decade ago, but with the anticipated release of ATP IV, it remains uncertain how and which risk models will be integrated into the recommendations. We sought to define the effects in the United States population of a transition from the FRS to the RRS for cardiovascular risk assessment.

**Methods:**

Using the National Health and Nutrition Examination Surveys, we assessed FRS and RRS in 2,502 subjects representing approximately 53.6 Million (M) men (ages 50–79) and women (ages 45–79), without cardiovascular disease or diabetes. We calculated the proportion reclassified by RRS and the subset whose LDL-C goal achievement changed.

**Results:**

Compared to FRS, the RRS assigns a higher risk category to 13.9% of women and 9.1% of men while assigning a lower risk to 35.7% of men and 2% of women. Overall, 4.7% of women and 1.1% of men fail to meet newly intensified LDL-C goals using the RRS. Conversely, 10.5% of men and 0.6% of women now meet LDL-C goal using RRS when they had not by FRS.

**Conclusion:**

In the U.S. population the RRS assigns a new risk category for one in six women and four of nine men. In general, women increase while men decrease risk. In conclusion, adopting the RRS for the 53.6 million eligible U.S. adults would result in intensification of clinical management in 1.6 M additional women and 2.10 M fewer men.

## Introduction

The National Cholesterol Education Panel’s Adult Treatment Panel III (ATP III) are the clinical guidelines used in the United States to identify and treat dyslipidemia for prevention of coronary heart disease (CHD). The ATP III guidelines endorse the calculation of a patient’s Framingham Risk Score (FRS) to assess ten-year CHD risk which helps physicians choose cholesterol goals based on cardiovascular risk level. [1] Clinician use of CHD risk scores in primary prevention of CHD results in reduction of CHD risk factors without additional clinical harm. [2] The ATP III guidelines however, are almost a decade old and over this time period new multivariate cardiovascular risk models have emerged. [3]–[8] Risk models differ in variables, definitions of endpoints and the population in which they were developed and validated. [3]–[6] (Table 1). The FRS, developed and validated in the Framingham cohort, has been the preferred risk model to determine ten year risk of CHD in the U.S. Recently, a newer multivariate risk model, the Reynolds Risk Score (RRS) was validated in both men and women. Compared to the FRS, the RRS variables do not include current blood pressure medication use and adds variables of hemoglobin A1C in female patients with diabetes, family history and high sensitive C-reactive protein (hs-CRP) in both men and women. [3]–[4] The two models also differ on their endpoints with FRS predicting death or myocardial infarction and RRS adding stroke and need for revascularization.

**Table 1:** Overview of Risk Models.

Risk Model	Variables	Endpoints	Validation Population
Framingham Risk Model	1. Age2. Gender3. Systolic blood pressure4. Total cholesterol5. High density lipoprotein6. Smoking7. Diabetes mellitus8. Blood pressure medications	Myocardial infarctionCoronary Death	United States
Reynolds Risk Model	1. Age2. Gender3. Systolic blood pressure4. Total cholesterol5. High density lipoprotein6. Smoking status7. Hemoglobin A1c (Women only)8. Family history9. High sensitivity C-Reactive protein	Myocardial infarctionCardiovascular death Ischemic strokeCoronary revascularization	United States
ASSIGN	1. Age2. Gender3. Systolic blood pressure4. Total cholesterol5. High density lipoprotein6. Cigarettes per day7. Diabetes mellitus8. Family history9. Scottish Index of Multiple Deprivation	Death from cardiovascular diseaseCoronary heart diseaseCerebrovascular diseaseCoronary revascularization	Scotland
SCORE	1. Age2. Systolic blood pressure3. Total cholesterol4. Total cholesterol/high density lipoprotein ratio	Fatal Cardiovascular events	Multiple Countries:Finland, Russia, Norway,United Kingdom, Denmark, Sweden, Belgium, Germany,Italy, France, Spain
QRISK2	1. Age2. Gender3. Total cholesterol/high density lipoprotein ratio4. Systolic blood pressure5. Smoking status6. Diabetes mellitus7. Family history8. Treated hypertension9. BMI10. Townsend Deprivation Score11. Self Assigned Ethnicity12. Rheumatoid arthritis13. Atrial fibrillation14. Chronic renal disease	Coronary Heart Disease(Myocardial infarction, Angina)StrokeTransient Ischemic Attack	United Kingdom

The RRS was developed and validated in the Physicians Health Study II (PHS-II) and the Women’s Health Study (WHS) reclassifying both men and women into higher or lower risk categories compared with a modified FRS with the same endpoint as the RRS. While the RRS reclassified subjects at each risk category level, the highest rate of reclassification occurred among moderate and moderate high risk women (44%) and men (20%) without diabetes. [3]–[4].

In 2009, the Canadian Cardiovascular Society released updated guidelines on the treatment of dyslipidemia and the prevention of cardiovascular disease, recommending the RRS as an alternate multivariate risk model to assign lipid goals. [9] In the United States, recent primary prevention performance guidelines and joint guidelines for assessment of cardiovascular risk in the asymptomatic patient recommend routine utilization of a multivariate risk model by clinicians and the RRS was considered an alternate to the FRS for absolute CHD risk calculation. [10]–[11].

As the ATP IV committee is currently meeting with the anticipated release of new cholesterol guidelines in 2012, it remains unclear how multivariate risk assessment will be integrated into risk assessment and furthermore, it remains unknown which risk model, if any, will be a preferred method for risk assessment. If a new multivariate risk model replaces the Framingham risk model previously integrated into ATP III, this may generate unknown effects by shifting risk distribution in the U.S. population. This risk reclassification will alter lipid goals, change clinical management and may lead to more diagnostic testing if this shift increases the intermediate risk population (FRS 6–20%). Because both the FRS and the RRS are ten year risk models developed and validated in U.S. based cohorts, and are used interchangeably in clinical practice for primary prevention of CHD we sought to further compare the clinical effects of using these models in a U.S. based population.

The purpose of this study is to analyze the population effects of replacing the Framingham Risk Score with the Reynolds Risk Score for cardiovascular risk assessment in U.S. adults eligible for primary prevention.

## Methods

The National Center for Health Statistics performs the National Health and Nutrition Examination Survey (NHANES) surveys in two-year increments to define the health and nutritional status of the United States population. All participants give informed written consent to participate in the NHANES. Data collection for NHANES was approved by the National Center for Health Statistics Research Ethics Review Board. Analysis of de-identified data from the survey is exempt from the federal regulations for the protection of human research participants. Analysis of restricted data through the National Center for Health Statistics Research Data Center is also approved by the National Center for Health Statistics Ethics Review Board. Because these are publicly available data files no institutional review board review is required. The surveys are comprised of a home health interview and a health examination that is performed in a mobile exam center (MEC). The NHANES utilizes complex, stratified, multistage sampling techniques based on demographic and geographical data, assigning subjects a weight such that the sum represents a statistical model of the entire civilian non-institutionalized United States population. Methods involve identification of primary sampling units, within which, clusters of households are identified with each person in the household screened for demographic characteristics. [12] The NHANES database has been used to develop national health standards, [13] assess disease prevalence, [14], [15], [16] identify risk factors for disease, [17], [18] and assess the health of the nation. [19] Detailed information on NHANES data collection is published and available at http://www.cdc.gov/nchs/nhanes.htm.

### Statistical Analysis

The NHANES data sets of 1999–2000 and 2001–2002 were downloaded and imported into Microsoft Excel (version 11.2 for Macintosh, Microsoft Corp, Redmond, Wash) and SAS version 9.2 (SAS Institute, Cary, NC). Framingham Risk Scores were calculated using formulas provided by the Framingham study. (Available at: http://www.framinghamheartstudy.org/risk/hrdcoronary.html). (Appendix S2) The Reynolds Risk Score was calculated using formulas from the developmental cohorts. [3]–[4] To account for the complex survey design of NHANES, PROC SURVEYMEANS in SAS was used to calculate standard errors using the Taylor Linearization method. [12] The 95% confidence intervals for estimated population parameters were calculated using the Wald method. Marginal homogeneity of RRS and FRS was assessed using the Wald test for the paired difference in mean ratings. A two-tailed p value of less than 0.05 was considered to be significant. In the present paper, we refer to both individual NHANES participant counts, presented as integers without ranges, and estimated population totals presented as millions with 95% confidence intervals.

### Inclusions/Exclusions

To match the population in which the RRS was developed in, we included all women ages 45–79 and men ages 50–79 free of CHD and diabetes mellitus (Table 2). The study population included 1,440 female subjects representing 33.5 million United States women and 1,062 male subjects representing 20.1 Million United States men.

**Table 2:** Inclusions/Exclusions from 1999–2002 NHANES Database.

	Subjects	Weighted Population*
MEC Group	19,759	278.7±8.3
Women <45Men <50	14,925	191.5±6.2
Age >79	724	8.0±0.6
MEC Women 45–79, Men 50–79	4110	79.2±3.2
Pregnant	4	0.07±0.04
Missing Blood Pressure Data	78	1.4±0.3
Missing Lipid Data	260	4.2±0.4
Diabetes Mellitus	708	9.8±0.7
ATP III Risk Equivalents(CHD, PVD, CVD)	534	9.7±0.7
Chemotherapy	24	0.4±0.1
Total Included Men 50–79	1062	20.1±0.9
Total Included Women 45–79	1440	33.5±1.7
Total Included Population	2502	53.6±2.4

* Population Weight in Millions ± SE.

Low density lipoprotein cholesterol (LDL-C) was calculated using the Friedewald equation [20] and was used to determine attainment of treatment goal. The corresponding non-HDL-C goals set forth by ATP III [1] was used for subjects with triglycerides > = 400 mg/dL, missing triglyceride data or with a fasting time <8 hours. C- reactive protein was measured in the NHANES using a latex-enhanced Dade Behring Nephelometer II Analyzer System (Dade Behring Diagnostics Inc., Somerville, NJ) [12].

### Definitions

#### High risk criteria

Because neither the FRS nor RRS are intended for use with subjects who have high-risk medical conditions, the following definitions were used within the NHANES data to exclude those subjects with ATP III risk equivalents including CHD, diabetes mellitus, peripheral vascular disease, and cerebrovascular disease. CHD is defined by subjects reporting they were told by a health care professional that they had a myocardial infarction, CHD or angina pectoris. Peripheral vascular disease is defined by subjects with an ankle brachial index of <0.9 in either leg. Cerebrovascular disease is defined by subjects reporting a history of stroke. Diabetes mellitus is defined by subjects being told by healthcare professional that they had diabetes mellitus, or they reported taking oral hypoglycemic or insulin, or they had an 8-hour fasting glucose ≥126 mg/dL or a random glucose ≥200 mg/dL. See Appendix S1 for NHANES item descriptions.

#### Risk categories and LDL cholesterol goals

Subjects underwent risk classification by FRS and RRS into the risk categories below. We defined low risk as a ten year risk <6%, consistent with the 34^th^ Bethesda Conference. [21]–[22] The moderate, moderate high and high risk categories risk values were defined consistent with current guidelines. [1] LDL-C goals are listed for each risk category and are derived using the aggressive optional clinical goals in the U.S. lipid guidelines. [23].

- High Risk: Score ≥20%; LDL-C <70 mg/dL
- Moderate High Risk: Score ≥10% and <20%; LDL-C <100 mg/dL
- Moderate Risk: Score ≥6% and <10%; LDL-C <130 mg/dL
- Low Risk: Score <6%; LDL-C <160 mg/dL

#### Clinically significant reclassification

Risk category reclassification changed a subject’s recommended LDL-C goals based on their new risk category. However, some subjects already met both their RRS and FRS goals, while others did not meet either goal. For both cases, cholesterol therapy decisions were not dependent on the score chosen which reduces the clinical significance of the reclassification. We defined a clinically significant reclassification as an instance where a subject’s LDL-C level was between the two different LDL-C goals. For these subjects, clinical management depended on the choice of using the RRS or the FRS. Thus, the decision to initiate or intensify lipid lowering therapy depended on which risk model is used. We further defined two sub-groups of these clinically significant reclassifications: a) those who met their RRS goal when they had not met their FRS goal (RRS < FRS) and b) those who did not meet their RRS goal when they previously met their FRS goal (RRS > FRS). For example, a 64-year-old-female smoker with a positive family history of CHD, a blood pressure of 130/80 mmHg, total cholesterol 190 mg/dL, HDL-C 46 mg/dL, LDL-C of 116 mg/dL and a hs-CRP of 3 mg/L has a FRS of 5.1% and a RRS of 12.5%. She meets the LDL-C goal for moderate risk of <130 mg/dL, but not the aggressive moderate high risk goal of <100 mg/dL.

## Results

### Subject Characteristics–Women

Descriptive characteristics of the 1440 female subjects representing 33.5±1.7 M women are listed in Table 3.

**Table 3:** Subject Characteristics.

	Women	Men
Age, median (IQR) yrs	54.2 (15.5)	57.9 (12.8)
LDL-C, median (IQR) mg/dL	129 (44.5)	134 (43.5)
Systolic Blood Pressure, median (IQR) mmHg	126.2 (26.1)	126.8 (20.6)
High sensitive C-Reactive Protein, median (IQR) mg/L	2.8 (4.7)	1.9 (2.9)
HDL, median, (IQR) mg/dL	57.2 (20.3)	44.5 (15.2)
Total cholesterol, median (IQR) mg/dL	214.1 (46.7)	209 (45.3)
Tobacco use, % (95% CI)	17.2 (14.5–19.8)	17.3 (14.2–20.5)
Premature familial atherosclerosis, % (95% CI)	15.4 (12.9–17.9)	7.7 (5.6–9.8)
Current use of blood pressure medications, % (95% CI)	26.2 (22.4–30)	21.8 (18.5–25)
Current use of lipid lowering medications, % (95% CI)	10.3 (8.6–11.9)	13.2 (10.4–16.1)

### Risk Reclassification Women

Analysis with the FRS resulted in 82% of the weighted population at low risk, 11.4% at moderate risk, 6% at moderate high risk and 0.6% at high risk. In contrast, using RRS, risk classifications tended to be more severe (p<0.0001) with 76% at low risk, 11% at moderate risk, 9.3% at moderate high risk, and 3% at high risk. (Table 4) In total, the RRS reclassified 15.9% of the weighted population into higher or lower categories, with 13.9% (95% CI, 11.6%–16.1%) having increased risk and 2.0% (95% CI, 1.1%–3.0%) having decreased risk category.

**Table 4:** Reynolds Risk Score Applied to Population of U.S. Women.

FRS	POPULATION	% OF POPULATION	RRS INCREASESRISK†	RRS DECREASESRISK†	NO LONGER ATLDL-C GOAL†	NEWLY ATLDL-C GOAL†
Low	27.44 M(24.71–30.18)	82%(79.5–84.5%)	8%(6.5–9.5%)	N/A*	3.5%(2.4–4.6%)	N/A*
Moderate	3.83 M(3.03–4.62)	11.4%(9.7–13.2%)	45%(35.4–54.8%)	9.8%(5–14.7%)	16%(7.3–24.5%)	4.6%(0.7–8.6%)
Moderate High	2.0 M(1.29–2.7)	6%(4–8%)	36%(21–51.4%)	13%(2.8–23%)	2.2%(0–6%)	0%
High	0.2 M(0.08–0.33)	0.6%(0.3–1%)	N/A*	22%(6.5–38%)	N/A*	0%
Total	33.5 M(30–36.9)	100%	13.9%(11.6–16.1%)	2.0%(1.1–3.0%)	4.7%(3.4–6.0%)	0.6%(0.2–1.2%)

* N/A: Risk category reclassification not possible in that direction.

(95% confidence intervals).

† Percentage is based on proportion of risk category experiencing risk category reassignment.

Analyzing the RRS reclassification by initial FRS risk category, 8% of weighted low risk women increased risk category; of the weighted moderate risk women, 45% increased and 10% decreased risk category; of weighted moderate high risk women 36% increased while 13% decreased risk category and 22% of the weighted high risk women decreased risk category. (Table 4).

Evaluating clinically significant reclassification in women, 4.7% (3.4–6.0%) weighted subjects formerly meeting LDL-C goal by the FRS no longer met goal by the RRS and may warrant an intensification of lipid management. In the other direction, just 0.6% (0.2–1.2%) of women previously not meeting their LDL-C goal by the FRS newly met goal by the RRS, making intensified lipid management unnecessary. [Table 2].

### Subject Characteristics–Men

Descriptive characteristics of the 1062 male subjects representing 20.1±0.9 M men are listed in Table 3.

### Risk Reclassification: Men

Application of the FRS to the eligible men resulted in 17.4% of the weighted population at low risk, 26.1% at moderate risk, 43.7% at moderate high risk and 12.8% at high risk. In contrast, using RRS, risk classifications tended to be more severe (p<0.0001) with 33.2% of the weighted population at low risk, 25.7% at moderate risk, 26.3% at moderate high risk, and 14.8% at high risk. (Table 5) Overall, the RRS reclassified 44.8% of the weighted male subjects with 9.1% (95% CI, 7.3%–10.9%) reclassified to a higher risk category and 35.7% (95% CI, 32.5%–38.8%) reclassified to a lower risk category.

**Table 5:** Reynolds Risk Score Applied to Population of U.S. Men.

FRS	POPULATION	% OF POPULATION	RRS INCREASESRISK†	RRS DECREASESRISK†	NO LONGER ATLDL-C GOAL†	NEWLY ATLDL-C GOAL†
Low	3.50 M(2.81–4.20)	17.4%(14.4–20.5%)	6.3%(1.8–10.8%)	N/A*	0.4%(0.0–1.1%)	N/A*
Moderate	5.24 M(2.28–6.21)	26.1%(22.2–30.0%)	6.1%(3.2–9.1%)	58.1%(50.9–65.4%)	2.7%(0.0–5.3%)	18.4%(12.9–24.0%)
Moderate High	8.78 M(7.72–9.84)	43.7%(39.7–47.6%)	14.7%(11.1–18.3%)	36.9%(30.9–43.0%)	0.8%(0.2–0.5%)	12.3%(7.9–16.6%)
High	2.57 M(2.10–3.03)	12.8%(10.7–14.9%)	N/A*	34.3%(26.5–42.1%)	N/A*	2.3%(0.0–4.9%)
Total	20.1 M(18.3–21.9)	100%	9.1%(7.3–10.9%)	35.7%(32.5–38.8%)	1.1%(0.3–1.9%)	10.5%(7.9–13.1%)

* N/A: Risk category reclassification not possible in that direction.

(95% confidence intervals).

† Percentage is based on proportion of risk category experiencing risk category reassignment.

Analyzing reclassification of men by FRS risk category, 6.3% of the weighted low risk increased risk category; of the moderate risk men, 6.1% increased and 58.1% decreased risk category; of moderate high risk men, 14.7% increased while 36.9% decreased risk category and of the high risk men, 34.3% decreased risk category.

Evaluating clinically significant reclassification in men, 1.1% of weighted subjects had met their LDL-C goal by the FRS and no longer met their LDL-C goal by the RRS. Conversely, 10.5% of men had not met their LDL-C goal by the FRS, but were at goal when using the RRS. [Table 5].

## Discussion

The principal finding of this study comparing RRS to FRS risk assessment across the U.S population, was that both the magnitude and direction of risk category reclassification differed between genders. The RRS reclassified 14% of women up and just 2% of women down. In contrast, 36% of men were reclassified down while 9% were reclassified up. Additionally, using our definition of clinical significance, a clinician addressing LDL cholesterol goals and therapy decisions, will find smaller totals of clinically significant reclassification, but a prominent imbalance of up and down reclassifications between genders. Analysis of clinically significant reclassification resulted in 10.5% of men and 0.6% of women who no longer qualified for more aggressive therapy, while 4.7% of women and just 1.1% of men should have increased therapy considered.

### Comparison to Development Cohorts

Comparing the present results from the NHANES with the results of the RRS category reclassification in the PHS-II and the WHS development cohorts, illustrate a similar direction of reclassification, but larger magnitude. In the PHS-II cohort, the RRS reclassified 11% of men to a decreased and 7% to an increased risk category compared to FRS. In the WHS, the RRS reclassified 6% of women to an increased and 2% to a decreased risk category. Compared to the NHANES, the WHS cohort shows a similar distribution of risk categories (85%, 10%, 4% 1% for risk groups lower to high respectively), and a similar increase in percent reclassification for the moderate (43% model A, 30% model B) and moderate high (43% model A, 29% model B) risk groups. Compared to the NHANES, the PHS-II cohort shows a risk distribution weighted more towards lower risk (28%, 34%, 29%, 10% lower to high risk) with a less prominent increase in reclassification of the moderate (20.4%) and moderate high (19.7%) groups (all PHS-II point estimates outside present study 95% CI.).

A likely explanation for differences in risk category reclassification between the present study and the PHS-II and the WHS may relate to model calibration. The FRS is known to miscalibrate risk in populations that differ from the Framingham cohort; however, the FRS has been successfully recalibrated for a specific population.[3]–[4], [24] Because the RRS and FRS models were developed in three different cohorts, this likely explains some of the variation seen when these models are applied to the NHANES population. Both the RRS and the FRS were developed and validated in homogenous ethnicities within the U.S. and future studies of broader populations across the U.S. that track event outcomes would allow better comparisons of the calibration, fit and generalizability of these risk models. The results of two recent mendelian randomized analyses may also impact the generalization of these risk models to other populations. These studies indicate that some genetic alterations in both C-reactive protein and HDL may not translate to a change in coronary events. [25]–[26].

### Clinically Significant Risk Reclassification in the U.S

In an effort to translate the differences between models to the likely impact seen in the clinic setting, we found that only a minority of subjects, who had been reclassified, would be given different clinical recommendations regarding cholesterol treatment. The ratio of reclassification to clinically significant reclassification varied from approximately 2∶1 (8%∶3.5%) for low risk women who increased risk category [Table 4], to 18∶1 (14.7%∶0.8%) for moderate high risk men for whom RRS decreases risk. While clinically significant reclassification is not a universally defined measure, we believe that this sort of comparison of different risk prediction methods would help illustrate the potential for actual change in therapy that clinicians could use to evaluate and choose their practice patterns.

This analysis is intended to give the practicing clinician perspective of the population effects of using either of these models. To illustrate this point, if a clinician using the FRS with a female patient with borderline LDL-C values, has decided to initiate treatment, it would be uncommon (4.6% of moderate risk women and 0.6% of all women) for the RRS to suggest a different treatment path. Other clinical considerations of the RRS include its narrower age range of validation, which requires physicians to maintain access to FRS for younger patients who need risk comprehensive assessment, the differences in the endpoints between the FRS and RRS, and the additional data needed to calculate a RRS. There may be an economic impact of risk reclassification, however this analysis was outside of the scope of this investigation. Recently an analysis of the Women’s Health Initiative Observational Cohort the RRS was found to be a better discriminator of clinical events compared with the FRS. [27] This finding may result in increased clinician use of the RRS for risk assessment in women. One further consideration pertaining to both the FRS and the RRS is that these are both ten year risk models and use of either model assigns a large portion of women to the low risk category (82% in FRS, 76% in RRS) and may not accurately define an individual’s lifetime risk. [28]–[29].

While percentages impacted by a change in practice may be small, the absolute number of individuals impacted can be large. The NHANES creates a statistical model of the entire civilian non-institutionalized United States population, allowing estimates of individuals with a potential change in clinical management. Of U.S. women, approximately 4.6 M (95% CI, 3.7–5.6 M) (13.7%) increased risk and 1.6 M (95% CI,1.1–2.1 M) (4.8% of total) of these women were now eligible for an intensification of clinical management. Conversely in the men, 7.2 M (95% CI, 6.1–8.3 M) (35.8%) decreased risk category with 2.1 M (95% CI, 1.5–2.7 M) (10.4%) men not meeting goal by the FRS would newly have met goal if the RRS is used. In the example above, even 0.6% of weighted women represented over 175,000 individuals. To get at this conundrum of identifying a small percentage of a large group, there may be benefit from studying the capacity of decision tools to assist primary care clinicians in their negotiation of these potentially complex practice patterns.

### Differences in Endpoints

Clinicians utilizing the RRS and the FRS interchangeably must be cognizant of the differing endpoints of each model. These differing endpoints may impact both risk reclassification and treatment decisions. The difference in the risk reclassification seen in men and women with these models may be due to the effect of specific endpoints and gender differences among these endpoints in the RRS. In the U.S., annually more women experience a stroke compared with men and women carry a higher lifetime stroke risk compared with men. [30] These differing endpoints may also impact treatment decisions as in the current U.S. guidelines, pharmacotherapeutic treatment of lipids for primary prevention of CHD is recommended based on current LDL-C and the corresponding ten year risk of CHD. When utilizing a model with more and different endpoints the clinician must be aware of the differing effects of pharmacotherapy. While use of HMG-CoA-reductase inhibitors for primary prevention of CHD is associated with 30% reduction in CHD mortality, the RRS also includes endpoints of ischemic stroke for which the use of HMG CoA-reductase inhibitors confer 14–21% relative risk reduction. [31]–[32].

### Limitations and Strengths

Some limitations of this cross-sectional study are inherent to the NHANES survey, including sampling and non-sampling errors. The questionnaires are self reported and thus subject to misunderstanding and recall bias.

A few definitions vary in their use. The definition of a positive family history differs slightly between the NHANES survey and the RRS. The NHANES defines a positive family history of CHD as a myocardial infarction or angina afflicting a parent, grandparent or sibling younger than 50 years of age regardless of gender. The RRS defines a positive family history as a parental history of myocardial infarction before the age of 60. We used the widely accepted 6% cutoff to separate low and moderate risk classification [21] whereas in the RRS development and validation a 5% cutoff was utilized. [3]–[4] We expect that definitional variation will change absolute numbers in risk categories, but are unlikely to substantially impact numbers of subjects crossing treatment thresholds.

We used the more aggressive LDL-C goal options provided in the guidelines (LDL-C <100 mg/dL for moderate high risk and <70 mg/dL for high risk) for the purpose of distinguishing LDL-C goals across risk categories. If the same goal is used for more than one category, for example using <130 mg/dL for both moderate and moderate high risk, a smaller proportion of subjects would meet our clinically significant reclassification definition. Due to the cross sectional nature of this analysis, appropriateness of reclassification is unable to be assessed through a method such as net reclassification index. [33] Finally, the generalizability of these findings outside the U.S. population may be limited as the NHANES are a population based statistical model of the entire civilian non-institutionalized United States population.

Among the study’s strengths, is the use of the NHANES dataset which utilizes complex, stratified, multistage sampling techniques based on demographic and geographical data, assigning subjects a weight such that the sum represents a statistical model of the entire civilian non-institutionalized United States population. This database is ideal for the assessment of the U.S. population effects when instituting a new multivariate risk model.

### Conclusions

It remains uncertain how the RRS should best be integrated into the United States guidelines for cardiovascular risk assessment in primary prevention of CHD. Nonetheless, clinicians have an increasing number of choices of which cardiovascular risk model to use in clinical practice and should be aware of the population effects of using a new multivariate risk model compared with the Framingham risk model. The choice of which risk model to use presents a challenge to the practicing U.S. clinician. A clinician transitioning to the RRS may be faced with a clinical dilemma, where the FRS would recommend lipid therapy initiation or intensification, but the RRS would recommend lipid goal relaxation. While some recent North American guidelines view these risk models as interchangeable, [9]–[11] this analysis illustrates the differing population effects between these two models in the U.S. population.

## Supporting Information

Appendix S1 (DOC)

Appendix S2 (DOC)
