# Supplementary material for: Women Up, Men Down: The Clinical Impact of Replacing the Framingham Risk Score with the Reynolds Risk Score in the United States Population
Source: PLoS One. 2012 Sep 12;7(9):e44347. doi: 10.1371/journal.pone.0044347 (PMC3440377; doi:10.1371/journal.pone.0044347)
Supplement: Appendix S2 — (DOC) [file pone.0044347.s002.doc]

Framingham Risk Score <http://www.framinghamheartstudy.org/risk/hrdcoronary.html>

| **Outcome: Hard CHD** | | | | | | |
| --- | --- | --- | --- | --- | --- | --- |
| **Gender** | **MEN (n=4261)** | |  | **Gender** | **WOMEN (n=5182)** | |
|  |  |  |  |  |  |  |
|  | **Cox** |  |  |  | **Cox** |  |
| **Independent** | **Parameter** |  |  | **Independent** | **Parameter** |  |
| **Variable** | **Coefficient** | **Means** |  | **Variable** | **Coefficient** | **Means** |
| Ln(AGE) | 52.009610 | 3.8926095 |  | Ln(AGE) | 31.764001 | 3.9213204 |
| Ln(TOTAL) | 20.014077 | 5.3441475 |  | Ln(TOTAL) | 22.465206 | 5.3628984 |
| Ln(HDL CHOL) | -0.905964 | 3.7731132 |  | Ln(HDL CHOL) | -1.187731 | 4.0146369 |
| Ln(SBP) | 1.305784 | 4.8618212 |  | Ln(SBP) | 2.552905 | 4.8376494 |
| TRT for HTN (SBP > 120) | 0.241549 | 0.1180474 |  | TRT for HTN (SBP > 120) | 0.420251 | 0.1428020 |
| CURRENT SMOKER | 12.096316 | 0.3356020 |  | CURRENT SMOKER | 13.075430 | 0.3236202 |
| Ln(AGE)*Ln(TOTAL) | -4.605038 | 20.8111562 |  | Ln(AGE)*Ln(TOTAL) | -5.060998 | 21.0557746 |
| Ln(AGE)*SMOKER† | -2.843670 | 1.2890301 |  | Ln(AGE)*SMOKER‡ | -2.996945 | 1.2519882 |
| Ln(AGE)*Ln(AGE) | -2.933230 | 15.2144965 |  |  |  |  |
|  |  |  |  |  |  |  |
| Average 10 Year Survival = | 0.940200 |  |  | Average 10 Year Survival = | 0.987670 |  |
|  |  |  |  |  |  |  |

Reynolds Risk Score Women:

Ridker PM, Buring JE, Rifai N, Cook NR. Development and validation of improved algorithms for the assessment of global cardiovascular risk in women: the Reynolds Risk Score. JAMA. Feb 14 2007;297(6):611-619.

(1-0.98634(exp((0.0799* age + 3.137*natural logarithm (systolic blood pressure)+0.18*natural logarithm(high sensitivity C-reactive protein)+1.382*natural logarithm (total cholesterol)-1.172*natural logarithm (high density lipoprotein cholesterol)+0.134*(hemoglobin A1c % if diabetic)+0.818*(if current smoker) +0.438*if family history) x 100%

Reynolds Risk Score Men:

Ridker PM, Paynter NP, Rifai N, Gaziano JM, Cook NR. C-reactive protein and parental history improve global cardiovascular risk prediction: the Reynolds Risk Score for men. Circulation. Nov 25 2008;118(22):2243-2251, 2244p following 2251.

(1-0.899^EXP((4.385 x natural logarithm(age) + 2.607 x natural logarithm(systolic blood pressure) + 0.963 x natural logarithm(total cholesterol) - 0.772 x natural logarithm(high-density lipoprotein) + 0.405 (if current smoker) + 0.102 x natural logarithm (high-sensitivity C-reactive protein) + 0.541 (if family history) x 100%
